# Head Acceleration Event Exposure during Pre-Season Training in Elite Men’s Rugby Union

**DOI:** 10.1186/s40798-026-01072-3

**Published:** 2026-07-15

**Authors:** Samuel Hudson, Gregory Roe, Matt Cross, Ben Jones, Simon Kemp, Keith Stokes

**Affiliations:** 1 Centre for Health and Injury and Illness Prevention in Sport, University of Bath, Bath, UK; 2 UK Collaborating Centre on Injury and Illness Prevention in Sport (UKCCIIS), University of Bath, Bath, UK; 3 Carnegie Applied Rugby Research (CARR) Centre, Carnegie School of Sport, Leeds Beckett University, Leeds, UK; 4 PREM Rugby, London, UK; 5 Division of Physiological Sciences, Department of Human Biology, Faculty of Health Sciences, University of Cape Town, Cape Town, South Africa; 6 School of Behavioural and Health Sciences, Faculty of Health Sciences, Australian Catholic University, Brisbane, QLD Australia; 7 Rugby Football League, Etihad Campus, Manchester, UK; 8 Rugby Football Union, Twickenham, UK; 9 Department of Epidemiology and Population Health, London School of Hygiene and Tropical Medicine, London, UK

**Keywords:** Head acceleration event, Instrumented mouthguard, Rugby union, Pre-season, Training

## Abstract

**Background:**

Head acceleration event (HAE) outcomes in elite men’s rugby union have been well described during matches and in-season training but have not been investigated during pre-season training.

**Objectives:**

Describe HAE incidence and magnitude during elite men’s pre-season rugby union training.

**Method:**

Data was collected from three English PREM Rugby clubs (44 training sessions and 53 players) during field-based training sessions using instrumented mouthguards (iMGs) throughout the pre-season training period before the 2025–26 season. Training drills were recorded using routinely tagged GPS data. The incidence of HAEs was calculated per player minute, for forwards and backs, and during training sessions during the first, middle, and final three-week phases of pre-season. A negative binomial generalised linear mixed model compared playing position, and phases of pre-season, expressed as incidence rate ratios (IRR).

**Results:**

The magnitude of HAEs were positively skewed. The mean incidence of HAEs ≥ 5 g and ≥ 0.4 krad/s^2^ during a pre-season training session was 0.08/min [95% CI 0.07–0.09] and equated to 5.09 ± 5.12 HAEs per player-training-session exposure. The mean incidence of HAEs ≥ 5 g and ≥ 0.4 krad/s^2^ for forwards was 0.10/min [0.08–0.11] and equated to 5.87 ± 5.51 HAEs per player-training-session exposure, compared to 0.05/min [0.04–0.07] and 3.22 ± 3.39 HAEs per player-training-session exposure for backs (IRR = 1.84 [1.02–3.31], *p* = 0.041). The mean HAE incidence for forwards during the first three weeks of pre-season was lower (0.05/min [0.03–0.08]) than the middle (0.09/min [0.07–0.11]) and final (0.13/min [0.11–0.15]) three weeks. The incidence of HAEs for backs was similar in the first (0.04/min [0.02–0.06]), middle (0.06/min [0.04–0.08] and final (0.06/min [0.04–0.08]) three weeks. The incidence of HAEs during the middle (IRR = 1.55 [1.14–2.11], *p* = 0.005) and final (IRR = 2.82 [2.06–3.85], *p* < 0.001) phases of pre-season was statistically significant.

**Conclusion:**

The incidence and magnitude of HAEs during pre-season is very low compared to matches and similar to in-season training. Future work should identify players who accumulate more HAEs in matches and training compared to the rest of the population, and why. This study completes a holistic understanding of HAE exposure throughout a season, which can be used to benchmark individual’s exposure against.

## Key Points

- The incidence of HAEs during elite men’s pre-season rugby union training is low compared to matches, and similarly to in-season training.
- The final six weeks of the pre-season, where contact training density increases, coincides with an increase in HAE incidence per forward player-training-session.
- This study completes a holistic understanding of population average HAE exposure during an elite rugby season. This benchmark data can be used to identify players who accumulate relatively more HAEs during matches and training.

## Introduction

Rugby union is a collision-based contact team sport with interspersed periods of high intensity exercise [1]. There are concerns about the potential negative long-term brain health outcomes from exposure to head acceleration events (HAEs). A HAE is defined as the head’s acceleration response to a short duration collision force elicited through direct head contact, or inertial loading via the body [2]. Estimated measures of repetitive head impacts have been shown to be associated with cognitive decline [3], elevated blood biomarkers of neurodegenerative pathology [4–6] and dementia and Parkinson’s disease [7, 8]. Instrumented mouthguards (iMGs) have been deployed globally in elite rugby matches and training to quantify HAEs in real-time [9]. As a result, it has been demonstrated that HAE exposure during in-season training is significantly lower than matches [10–13].

Currently, HAE exposure during the pre-season period is unknown, although pre-season is associated with a higher risk of training concussion compared to in-season [14]. Pre-season typically follows a 5 to 7 week period of ‘off-season’, whereby players are not exposed to any contact. During pre-season, the purpose is to prepare players physically, technically and tactically for competition. After an initial period focusing on physical conditioning and skill development during pre-season, the volume of contact training increases [15]. Exposure to HAEs during the reintroduction of periodised contact training in elite men’s rugby is unknown and the incidence of HAEs might differ during the pre-season compared to the in-season, where HAE exposure was low during contact training [13]. The structure of pre-season contact training, combined with there being no requirements to be prepared for competition during this period, might lead to differences in HAE exposure observations. As a modifiable environment where coaches and practitioners can adapt the intensity of contact activities, HAE reduction strategies might be aimed at training [16]. Therefore, the aim of this study was to describe the incidence of HAEs in training sessions during an elite men’s rugby pre-season and during different training phases within the pre-season period.

## Method

### Study Design and Participants

A prospective observational cohort study was conducted with three men’s PREM Rugby teams, the highest level of rugby in England, during the 2025/2026 pre-season. Data was collected during 44 first-team training sessions. Ethics approval was granted by the Biomedical Sciences Research Ethics Committee (EP 20/21 088) at the University of Bath. Across the three teams, 127 players provided fully informed consent to take part in the study.

### Data Collection

#### Instrumented Mouthguards

In the elite game globally, as part of the head injury assessment (HIA) process iMGs have been mandated by World Rugby, the international governing body for the sport. As such, PREM Rugby teams are equipped with Prevent Biometrics iMGs (Minneapolis, MN, USA). Players wore custom fitted iMGs, that were moulded using 3D digital dental scans by an experienced dentist. Players were given the option by World Rugby of alternatively wearing a retainer version, which is thinner and smaller than the mouthguard. The mouthguard and retainer versions have the same instrumented specifications and performance but lower orofacial injury protection. Players had the option of wearing either the iMG or the retainer version. The iMGs were fitted with an accelerometer and gyroscope that sampled at 3.2 kHz and measurement ranges of ± 200 g and ± 35 rad/s. The validity of iMGs have been established, both in-lab and on-field [17–19] and met the World Rugby performance specifications.

The iMG continuously monitored head kinematics on the three planes of motion (sagittal, frontal, transverse) whilst coupled to the upper dentition, recognised through an embedded infrared proximity sensor. When linear acceleration head kinematics along one of its axes exceeded 8 g in magnitude, the iMG recorded a sensor acceleration event, which approximated the true in-vivo HAE. Data was recorded from 10 ms pre-trigger, to 40 ms post-trigger from which, resultant peak linear acceleration (PLA) (*g*) and peak angular acceleration (PAA) (krad/s^2^) were calculated. Tangential effects occur whereby the forces recorded at the iMG location differ to that at the head centre of gravity due to the iMG rotating faster around the centre of mass [20]. Therefore, linear head kinematics were transformed to the head centre of gravity at the 50th percentile of men’s head centre of gravity. The iMGs can record HAEs lower than 8 g when the 6-degree-of-freedom kinematics are transformed from the iMG location to the head centre of gravity. A 4-pole, zero-phase, low-pass Butterworth filter was applied. The HAEs were categorised by signal quality as minimal (class 0; *n* = 1,168), moderate (1; *n* = 73) or severe noise (2; *n* = 37), based on Prevent Biometrics’ in-house algorithm. These categories corresponded to cut-off frequencies of 200, 100, and 50 for classes 0, 1 and 2, respectively. To ensure the accuracy of HAE magnitudes, class 2 (severe noise) sensor acceleration events were excluded. Any HAE < 5 g and < 0.4 krad/s^2^ were excluded from the analysis, based on previous recommendation [10]. This threshold has a positive predictive value of 0.99 (95% CI 0.97–1.00) for removing non-contact HAEs [9] and has been used in other studies assessing HAE exposure in training [11–13].

Each club had a practitioner who routinely oversaw iMG deployment and data collection within their club. This staff member acted as a gatekeeper between the researcher and team. Practitioners were asked to distribute iMGs before the start of all field-based training sessions, and to remind players of the study and to encourage them to wear their iMG during all training activities, regardless of the prescribed level of contact training and perceived risk of HAE exposure and orofacial injury. Both HAE, and iMG time-on-teeth data, recorded by the infrared proximity sensor, was downloaded from the Prevent Biometrics portal into a Microsoft excel (version 16.102.1).csv file.

#### Training Exposure

Catapult (Catapult Sports, Melbourne, Australia) GPS units are employed league-wide by PREM Rugby teams, and their use in training is standard practice. For all field-based training sessions, practitioners defined all drills using GPS, tagging their start, end, and duration (HHMMSS). The date of the training session was also recorded (dd/mm/yyyy). This information for each training drill in each field-based training session during the pre-season was shared as a.csv file in Microsoft Excel. No GPS running metrics were shared.

The iMG time-on-teeth data was used to identify players that wore their iMG during training drills. The duration of a training drill was defined as an activity that starts and ends through coach instruction, or when the intensity of contact or nature of the activity changes. It was assumed that a player had worn their iMG for their full involvement in training drills if they had at least one period of iMG time-on-teeth during the training drill; previous research has demonstrated that players typically wear their iMG for the entirety of their drill involvements [11, 13]. Using iMG time-on-teeth time periods relative to GPS tagged training drills to quantify training exposure has also been used in research investigating HAEs in rugby league training [12].

A training session was defined as the sum of all drill durations within a scheduled session. At the training-session level, player exposures were only included if they wore their iMG during all contact training activities reported that day. This was to ensure that all potential HAEs were recorded. Each player-training-session exposure was a unique observation to capture differences in training session structure and content. Players with incomplete iMG time-on-teeth during all contact training activities were therefore excluded from the analysis.

As the pre-season was nine weeks long, player-training-session exposures were organised into the first, middle, and last 3 weeks at a team rather than player level. This was to allow for comparison between different phases of the pre-season.

### Statistical Analysis

The HAE, time-on-teeth, and GPS data were read into R (version 4.5.1) for analysis in RStudio (version 2025.09.1 + 401). The incidence of HAEs was calculated per player minute by summing the absolute count of HAEs and dividing by minutes of training exposure. The HAE incidence was calculated for all HAEs at the lowest threshold (≥ 5 g and ≥ 0.4 krad/s^2^) and at PLA and PAA thresholds in intervals of 5 g and 0.5 krad/s^2^, up to 25 g and 2.5 krad/s^2^ based on previous studies [13], respectively. Training-session observations belonging to each resampled player were retained together. Data wrangling of time-on-teeth data and GPS data was used to identify all player-drill and player-training-session exposures that occurred during the pre-season. The HAE incidence was calculated for each player in each training session. The HAE incidences for each player-training-session were then organised into the three phases of the pre-season to calculate the differences between them. Overall incidence rates for training sessions and phases in the pre-season period were calculated by averaging the HAE incidence rates for each exposure observation, to appreciate inter-player variability in HAE incidence. These were then averaged to calculate mean incidence [9, 13, 21]. To assess the distribution of HAE incidence between players, the interquartile range was also calculated. This was due to inter-player variability in HAE incidence during in-season training sessions being demonstrated in the same cohort [13].

A Poisson generalised linear mixed model (GLMM) was fitted using the lme4 package [22] but demonstrated overdispersion (X_2_ = 1.83, *p* < 0.001). Therefore, a negative binomial GLMM was fitted using the *glmmTMB* package [23] to compare player-training-session HAE incidence. The negative binomial GLMM showed better model fit than the Poisson GLMM (Akaike Information Criterion (AIC) = 1,228 and 1,313, respectively). Fixed effects included playing position and pre-season phase, with a random intercept for player. A position-phase interaction term was omitted due to a likelihood ratio test not significantly improving the model (X(2)^2^ = 2.89, *p* = 0.236). Fixed-effect estimates are reported as incidence rate ratios, with 95% CIs derived by exponentiating the model coefficients, and statistical significance set at *p* ≤ 0.05.

## Results

**Fig. 1:**
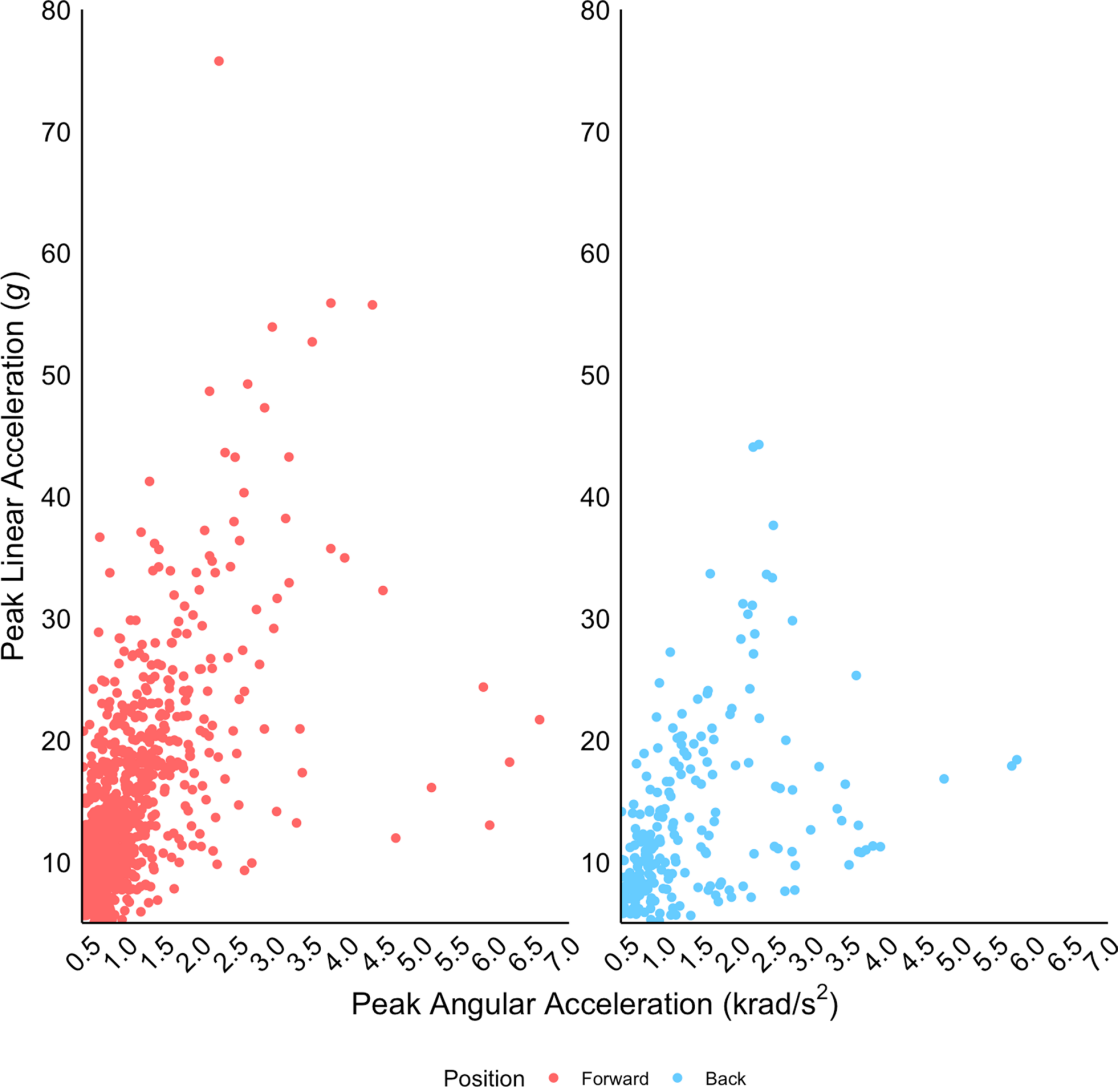
The magnitude of head acceleration events during pre-season training for forwards and backs

**Fig. 2:**
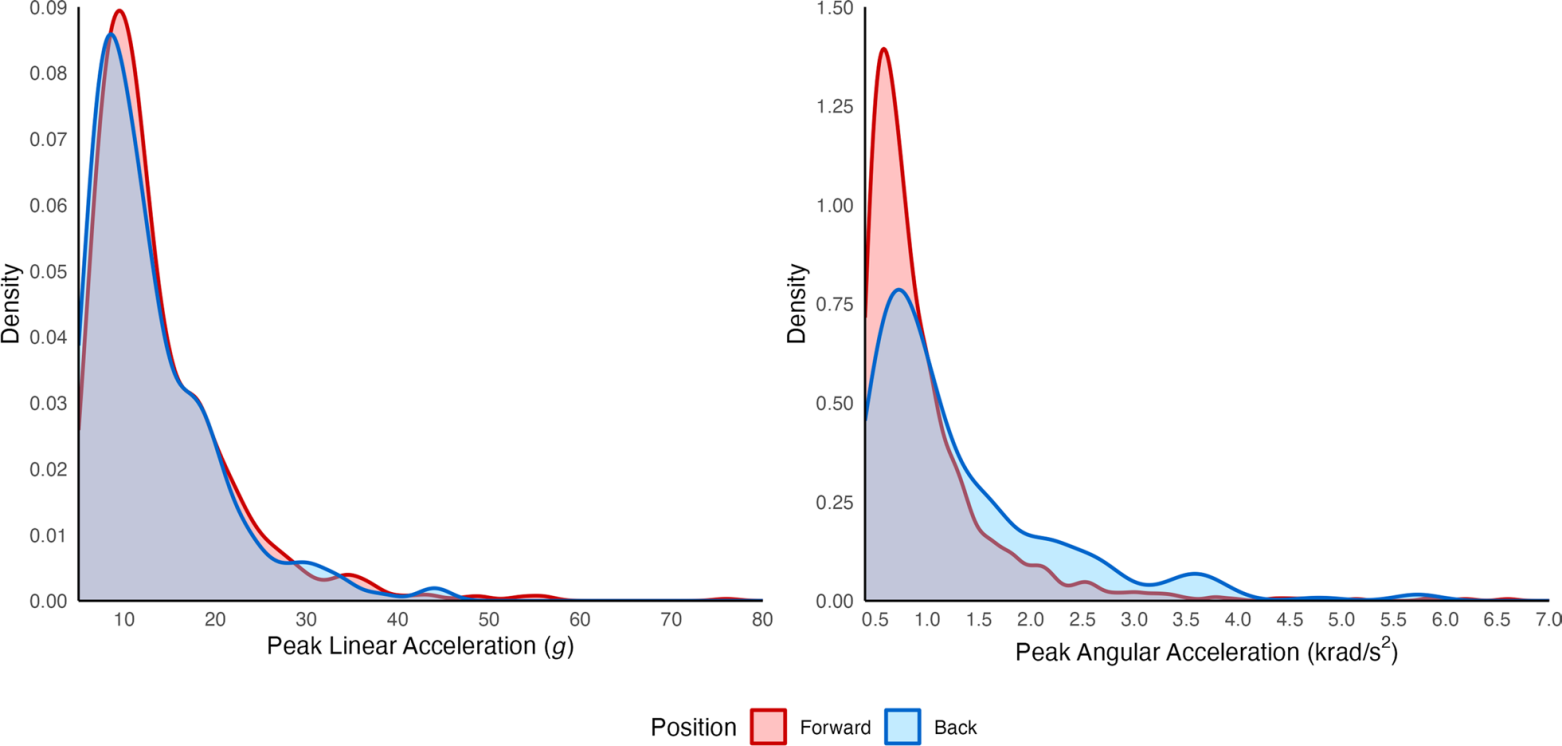
Density plot showing the distribution of the PLA and PAA magnitudes of HAEs for forwards and backs during the pre-season

During the pre-season, 53 players (35 forwards and 18 backs) wore their iMG and provided at least one exposure sample (42% of consenting players), during 44 training sessions (mean duration = 62.1 ± 21.2 min) and 244 player-training-session exposures (172 forward [4.9 ± 4.2 observations per player], 72 back [4.0 ± 2.4]). There were 1,241 HAEs ≥ 5 g and ≥ 0.4 krad/s^2^ (Fig. 1) recorded, which equated to 5.09 ± 5.12 HAEs per player-training-session exposure. There were 21 HAEs ≥ 25 g and ≥ 2.5 krad/s^2^ (1.7%) recorded, which equated to 0.11 ± 0.01 HAEs per player-training-session exposure. Forwards recorded 1,009 HAEs ≥ 5 g and ≥ 0.4 krad/s^2^ (Fig. 1), which equated to 5.87 ± 5.51 HAEs per player-training-session exposure. The magnitude of forward HAEs were positively skewed (Fig. 2), with 327 HAEs (32.4%) < 10 g and 693 HAEs (68.7%) < 1.0 krad/s^2^. The magnitude of back HAEs were positively skewed (Fig. 2), with 89 HAEs (38.4%) < 10 g and 121 HAEs (52.2%) < 1.0 krad/s^2^. There were 19 HAEs ≥ 25 g and ≥ 2.5 krad/s^2^ (1.9%) for forwards, which equates to 0.13 ± 0.36 per player-training-session exposure. Backs recorded 232 HAEs ≥ 5 g and ≥ 0.4 krad/s^2^ (Fig. 1), which equates to 3.22 ± 3.39 HAEs per player-training-session exposure. There were 2 HAEs ≥ 25 g and ≥ 2.5 krad/s^2^ (0.9%) for backs, which equates to 0.05 ± 0.23 per player-training-session exposure.

**Fig. 3:**
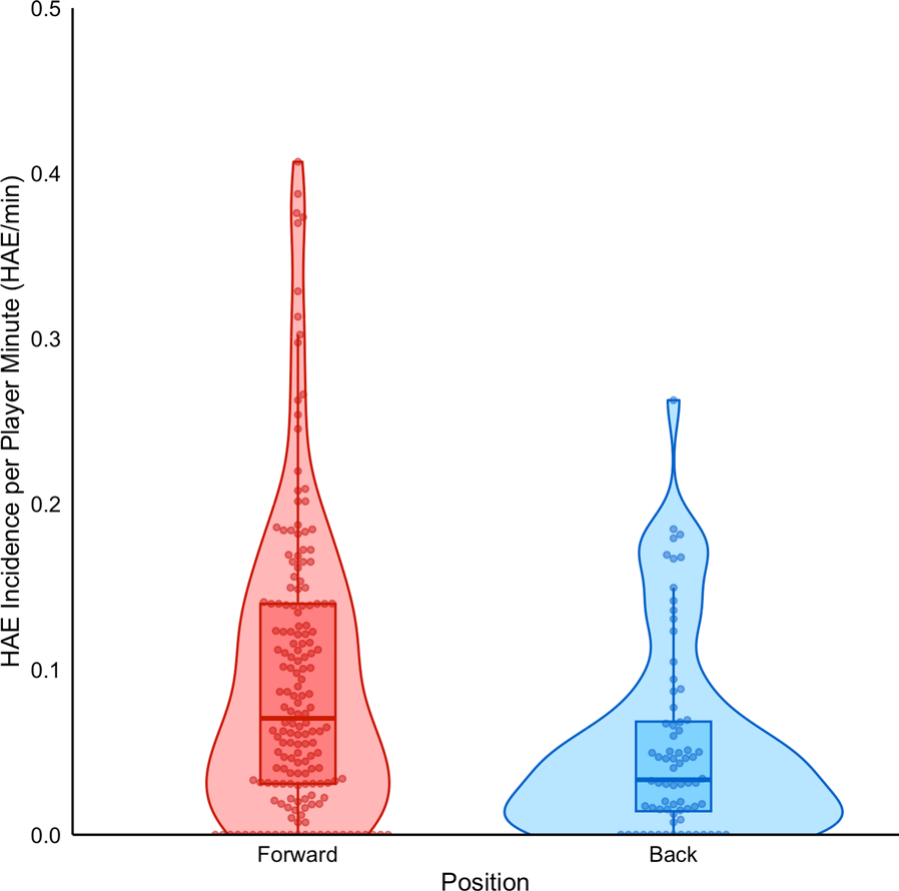
Violin plot of ≥ 5 g and ≥ 0.4 Krad/s^2^ head acceleration event incidence per player minute for forwards and backs during pre-season training sessions. Individual data points within the violin plots represent unique player training-session-exposures

The mean incidence of HAEs ≥ 5 g and ≥ 0.4 krad/s^2^ per player minute in a pre-season training session was 0.08/min [95% CI 0.07–0.09] (Table 1). The interquartile range of the incidence of HAEs ≥ 5 g and ≥ 0.4 krad/s^2^ per player minute in a pre-season training session was 0.11/min [0.09–0.12]. The mean incidence of HAEs ≥ 5 g and ≥ 0.4 krad/s^2^ per player minute in a pre-season training session for forwards was 0.10/min [0.08–0.11], and 0.05/min [0.04–0.07] for backs. The interquartile range of the incidence of HAEs ≥ 5 g and ≥ 0.4 krad/s^2^ per player minute in a pre-season training session was 0.11/min [0.09–0.13] for forwards and 0.05/min [0.04–0.07] for backs (Table 2; Fig. 3).

**Table 1:** The incidence of HAEs per player minute (and 95% confidence intervals) at different peak linear (PLA) and peak angular acceleration (PAA) thresholds for all players, and for forwards and backs, during a pre-season training session, and during different periods in the pre-season

	Peak linear acceleration (PLA; *g*)					Peak angular acceleration (krad/s^2^)				
	≥ 5	≥ 10	≥ 15	≥ 20	≥ 25	≥ 0.4	≥ 1.0	≥ 1.5	≥ 2.0	≥ 2.5
Training	0.08 [0.07–0.09]	0.06 [0.05–0.06]	0.03 [0.02–0.03]	0.01 [0.01–0.02]	0.01 [0.01–0.01]	0.08 [0.07–0.09]	0.03 [0.02–0.03]	0.01 [0.01–0.02]	0.01 [0.01–0.01]	0.00 [0.00–0.01]
Forward	0.10 [0.08–0.11]	0.06 [0.05–0.07]	0.03 [0.03–0.04]	0.02 [0.01–0.02]	0.01 [0.01–0.01]	0.10 [0.08–0.11]	0.03 [0.03–0.04]	0.01 [0.01–0.02]	0.01 [0.00–0.01]	0.00 [0.00–0.01]
Back	0.05 [0.04–0.07]	0.03 [0.02–0.04]	0.02 [0.01–0.02]	0.01 [0.00–0.01]	0.00 [0.00–0.01]	0.05 [0.04–0.07]	0.03 [0.02–0.04]	0.02 [0.01–0.02]	0.01 [0.01–0.02]	0.01 [0.00–0.01]
Pre-season period										
First 3 weeks	0.06 [0.03–0.08]	0.04 [0.02–0.05]	0.02 [0.01–0.03]	0.01 [0.00–0.01]	0.01 [0.00–0.01]	0.06 [0.03–0.08]	0.02 [0.01–0.04]	0.01 [0.00–0.03]	0.01 [0.00–0.02]	0.00 [0.00–0.01]
Forward	0.05 [0.03–0.08]	0.05 [0.02–0.07]	0.02 [0.01–0.04]	0.01 [0.01–0.02]	0.01 [0.00–0.01]	0.07 [0.04–0.10]	0.02 [0.01–0.04]	0.01 [0.00–0.02]	0.01 [0.00–0.01]	0.00 [0.00–0.01]
Back	0.04 [0.02–0.06]	0.02 [0.01–0.03]	0.01 [0.00–0.01]	0.00 [0.00–0.01]	0.00 [0.00–0.00]	0.04 [0.01–0.07]	0.02 [0.00–0.06]	0.02 [0.00–0.06]	0.01 [0.00–0.02]	0.00 [0.00–0.02]
Middle 3 weeks	0.08 [0.06–0.09]	0.07 [0.06–0.13]	0.03 [0.03–0.06]	0.02 [0.01–0.03]	0.01 [0.01–0.02]	0.11 [0.09–0.19]	0.02 [0.02–0.04]	0.01 [0.01–0.02]	0.01 [0.00–0.01]	0.00 [0.00–0.01]
Forward	0.09 [0.07–0.11]	0.09 [0.07–0.16]	0.04 [0.03–0.07]	0.02 [0.02–0.04]	0.01 [0.01–0.02]	0.13 [0.10–0.23]	0.02 [0.02–0.04]	0.01 [0.01–0.02]	0.01 [0.00–0.01]	0.00 [0.00–0.01]
Back	0.06 [0.04–0.08]	0.03 [0.02–0.08]	0.02 [0.01–0.04]	0.01 [0.00–0.01]	0.00 [0.00–0.01]	0.05 [0.04–0.12]	0.02 [0.01–0.05]	0.01 [0.01–0.02]	0.01 [0.00–0.03]	0.01 [0.00–0.03]
Final 3 weeks	0.11 [0.09–0.12]	0.07 [0.04–0.08]	0.04 [0.02–0.04]	0.02 [0.01–0.02]	0.01 [0.00–0.01]	0.11 [0.06–0.12]	0.04 [0.02–0.04]	0.02 [0.01–0.02]	0.01 [0.01–0.01]	0.00 [0.00–0.01]
Forward	0.13 [0.11–0.15]	0.09 [0.05–0.10]	0.04 [0.03–0.05]	0.02 [0.01–0.03]	0.01 [0.01–0.01]	0.13 [0.07–0.15]	0.04 [0.02–0.05]	0.02 [0.01–0.02]	0.01 [0.00–0.01]	0.00 [0.00–0.00]
Back	0.06 [0.04–0.08]	0.04 [0.02–0.07]	0.02 [0.01–0.04]	0.01 [0.00–0.03]	0.00 [0.00–0.01]	0.06 [0.03–0.10]	0.03 [0.01–0.04]	0.02 [0.01–0.03]	0.01 [0.01–0.02]	0.01 [0.00–0.01]

**Table 2:** The interquartile range of the incidence of HAEs per player minute (and 95% confidence intervals) at different peak linear (PLA) and peak angular acceleration (PAA) thresholds for all players, and for forwards and backs, during a pre-season training session, and during different periods in the pre-season

	Peak linear acceleration (PLA; *g*)					Peak angular acceleration (krad/s^2^)				
	≥ 5	≥ 10	≥ 15	≥ 20	≥ 25	≥ 0.4	≥ 1.0	≥ 1.5	≥ 2.0	≥ 2.5
Training	0.11 [0.09–0.12]	0.09 [0.07–0.15]	0.05 [0.03–0.08]	0.02 [0.02–0.04]	0.01 [0.01–0.02]	0.11 [0.09–0.12]	0.06 [0.03–0.09]	0.03 [0.02–0.04]	0.02 [0.01–0.02]	0.01 [0.00–0.01]
Forward	0.11 [0.09–0.13]	0.14 [0.06–0.22]	0.07 [0.03–0.12]	0.03 [0.02–0.07]	0.02 [0.01–0.04]	.11 [0.09–0.13]	0.08 [0.03–0.12]	0.04 [0.01–0.05]	0.02 [0.01–0.03]	0.01 [0.01–0.02]
Back	0.05 [0.04–0.07]	0.04 [0.02–0.09]	0.02 [0.01–0.07]	0.01 [0.01–0.03]	0.01 [0.00–0.01]	0.05 [0.04–0.07]	0.04 [0.01–0.07]	0.02 [0.01–0.04]	0.02 [0.00–0.02]	0.01 [0.01–0.02]
Pre-season period										
First 3 weeks	0.07 [0.05–0.11]	0.05 [0.04–0.06]	0.02 [0.02–0.04]	0.02 [0.00–0.02]	0.00 [0.00–0.02]	0.07 [0.05–0.11]	0.02 [0.02–0.04]	0.02 [0.00–0.02]	0.00 [0.00–0.02]	0.00 [0.00–0.00]
Forward	0.08 [0.05–0.15]	0.06 [0.03–0.10]	0.04 [0.02–0.06]	0.02 [0.02–0.03]	0.00 [0.00–0.02]	0.08 [0.05–0.15]	0.04 [0.02–0.05]	0.02 [0.00–.03]	0.00 [0.00–0.02]	0.00 [0.00–0.01]
Back	0.06 [0.03–.11]	0.04 [0.00–0.05]	0.02 [0.00–0.02]	0.00 [0.00–0.02]	0.00 [0.00–0.02]	0.06 [0.03–.11]	0.02 [0.00–0.05]	0.02 [0.00–0.02]	0.01 [0.00–0.02]	0.00 [0.00–0.00]
Middle 3 weeks	0.10 [0.08–.12]	0.08 [0.05–0.09]	0.03 [0.03–0.04]	0.02 [0.01–0.02]	0.00 [0.00–0.02]	0.10 [0.08–.12]	0.04 [0.04–0.06]	0.02 [0.01–0.02]	0.00 [0.00–0.02]	0.00 [0.00–0.00]
Forward	0.10 [0.09–.14]	0.07 [0.06–0.09]	0.04 [0.03–0.05]	0.02 [0.02–0.03]	0.00 [0.00–0.02]	0.10 [0.09–.14]	0.03 [0.03–0.05]	0.02 [0.01–0.02]	0.00 [0.00–0.02]	0.00 [0.00–0.00]
Back	0.05 [0.03–0.13]	0.05 [0.02–0.10]	0.02 [0.00–0.04]	0.00 [0.00–0.02]	0.00 [0.00–0.01]	0.05 [0.03–0.13]	0.05 [0.02–0.07]	0.02 [0.01–0.04]	0.01 [0.00–0.03]	0.00 [0.00–0.02]
Final 3 weeks	0.11 [0.09–0.14]	0.08 [0.07–0.10]	0.06 [0.04–0.08]	0.03 [0.02–0.05]	0.02 [0.00–0.02]	0.11 [0.09–0.14]	0.06 [0.04–0.07]	0.03 [0.02–0.03]	0.02 [0.01–0.02]	0.00 [0.00–0.02]
Forward	0.12 [0.08–0.16]	0.09 [0.06–0.11]	0.07 [0.04–0.08]	0.04 [0.02–0.05]	0.02 [0.01–0.03]	0.12 [0.08–0.16]	0.06 [0.04–0.08]	0.03 [0.02–0.04]	0.02 [0.00–0.02]	0.00 [0.00–0.02]
Back	0.07 [0.03–0.14]	0.05 [0.02–0.08]	0.03 [0.01–0.05]	0.02 [0.00–0.02]	0.00 [0.00–0.01]	0.07 [0.03–0.14]	0.05 [0.02–0.06]	0.02 [0.02–0.03]	0.02 [0.01–0.03]	0.01 [0.00–0.02]

**Fig. 4:**
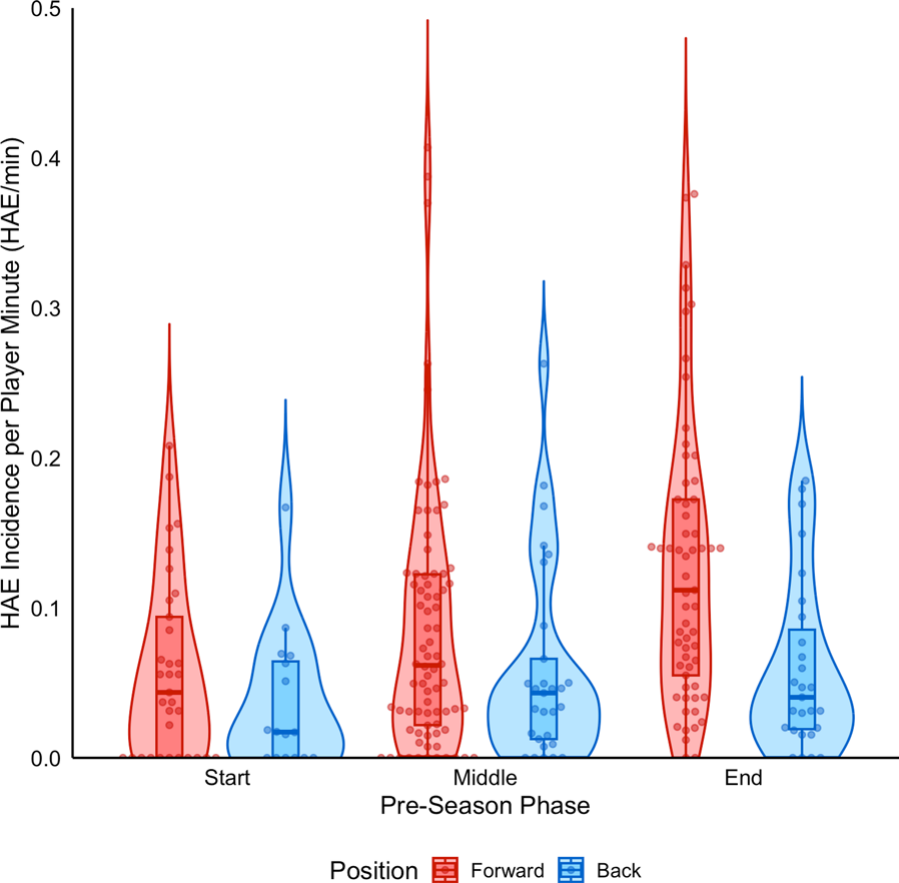
Violin plot of ≥ 5 g and ≥ 0.4 Krad/s^2^ head acceleration event incidence per player minute during pre-season training sessions for forwards and backs during the first, middle, and final three weeks phases of the pre-season training period. Individual data points within the violin plots represent unique player training-session-exposures

The mean incidence of HAEs ≥ 5 g and ≥ 0.4 krad/s^2^ per player minute was 0.06/min [0.03–0.08] during the first three weeks of pre-season, 0.08/min [0.06–0.09] during the middle three weeks, and 0.11/min [0.09–0.13] in the final three weeks (Table 1). The mean incidence of HAEs ≥ 5 g and ≥ 0.4 krad/s^2^ per player minute was 0.05/min [0.03–0.08] for forwards during the first three weeks of pre-season, 0.09/min [0.07–0.11] during the middle three weeks, and 0.13/min [0.11–0.15] during the final three weeks. The mean incidence of HAEs ≥ 5 g and ≥ 0.4 krad/s^2^ per player minute was 0.04/min [0.02–0.06] for backs during the first three weeks of pre-season, 0.06/min [0.04–0.08] during the middle three weeks, and 0.06/min [0.04–0.08] during the final three weeks. The interquartile range of the incidence of HAEs ≥ 5 g and ≥ 0.4 krad/s^2^ per player minute was 0.07/min [0.05–0.11] in the first three weeks of pre-season, 0.10/min [0.08–0.12] in the middle three weeks, and 0.11/min [0.09–0.14] in the final three weeks (Fig. 4; Table 2). The interquartile range of the incidence of HAEs ≥ 5 g and ≥ 0.4 krad/s^2^ per player minute was 0.08/min [0.05–0.15] for forwards during the first three weeks of pre-season, 0.10/min [0.09–0.14] during the middle three weeks, and 0.12/min [0.08–0.16] during the final three weeks. The interquartile range of the incidence of HAEs ≥ 5 g and ≥ 0.4 krad/s^2^ per player minute was 0.06/min [0.03–0.11] for backs during the first three weeks, 0.05/min [0.03–0.13] during the middle three weeks, and 0.07/min [0.03–0.14] during the final three weeks.

The negative binomial rate of HAEs for forwards was approximately twice as high than backs during pre-season training (incidence rate ratio (IRR) = 1.84 [1.02 to 3.31], *p* = 0.041) (Table 3). Compared to the start of pre-season, HAE rates were higher in the middle (RR = 1.55 [1.14 to 2.11], *p* = 0.005) and final phases (RR = 2.82 [2.06 to 3.85], *p* < 0.001) of pre-season. Player-level random effects demonstrated moderate-to-large variance (intracalss correlation coefficient (ICC) = 0.23).

**Table 3:** Incidence rate ratios (IRR) with significance set at *p* ≤ 0.05 and 95% CIs between playing position, and pre-season phases in training-related HAEs

Interaction		Incidence rate ratio (IRR)	95% CI Lower	95% CI Upper	*p*-value
Position	Forward vs Back	1.84	1.02	3.31	0.041*
Pre-season phase	Start vs Middle	1.55	1.14	2.11	0.005*
	Start vs Final	2.82	2.06	3.85	< 0.001*

^*^Indicates statistical significance

## Discussion

This is the first study to describe the incidence of HAEs during pre-season training in elite men’s rugby. The incidence of all HAEs (≥ 5 g and ≥ 0.4 krad/s^2^) and higher magnitude HAEs (> 25 g and 2.5 krad/s^2^) during pre-season training sessions was very low compared to matches [9] and was similarly to in-season training sessions [11–13]. The magnitude of HAEs were positively skewed, similarly to in-season training [13]. However, a slightly higher proportion of HAEs during in-season training compared to pre-season were < 10 g (38.1% vs 32.4% for forwards, 42.3% and 38.4% for backs, respectively) and were similar for forwards < 1.0 krad/s^2^ (67.3% vs 68.7, respectively), and more prominent for backs (64.9% and 52.2% respectively) [13]. On average, forwards and backs typically experienced 6 and 3 HAEs at the lowest magnitude (≥ 5 g and ≥ 0.4 krad/s^2^) per pre-season training session, respectively. Forwards observed approximately two-times higher mean HAE incidence rates than backs, and their mean HAE incidence increased in the middle and final three weeks of the pre-season increased. However, it was still relatively low during these periods compared to matches [10]. The mean incidence of HAEs for backs meanwhile remained consistently low throughout the pre-season.

The incidence of concussions during pre-season training has previously been reported to be approximately twice as high as during in-season training within the same cohort as the present study [14]. In contrast, the incidence of HAEs observed in pre-season during the present study was similar to values reported during the in-season [11–13] but increased notably in the middle (IRR = 1.55 [1.14 to 2.11], and particularly final phase (IRR = 2.82 [2.06 to 3.85]) of the pre-season. Furthermore, despite forwards experiencing an average HAE incidence during high-contact periods of pre-season training that was roughly twice that of backs, the overall incidence of concussion does not differ between the positional groups [14]. This suggests that even though forwards accumulate greater HAE exposure during pre-season than backs, and mean HAE incidence in pre-season is comparable to in-season training, concussion risk remains similar between positions and is elevated in pre-season relative to in-season periods. Players who exhibit a greater baseline concussion risk during training [14] may possess intrinsic or behavioural characteristics that contribute to the accumulation of more HAEs than is typical for their position. The present study demonstrated inter-player variability in the incidence of HAEs (ICC = 0.23), particularly for forwards during the middle and end periods of pre-season (Fig. 4) where contact training volume and intensity likely increases. While this study did not record concussions or the mechanisms of the HAEs recorded, it could be hypothesised that the players who accumulate more HAEs during the pre-season than the population average might be the same as those who demonstrate increased training concussion risk. Identifying these players and understanding the underlying reasons for their increased HAE outcomes should be a priority for future research.

During the middle phase of pre-season, training begins to replicate match demands and as such, the intensity and nature of contact training shift accordingly [24]. This coincided with a 55% increase in HAE incidence compared to the first phase. Contact conditioning activities aim to replicate the highest intensity periods during competition [24, 25] and skill execution under fatigue [26] which is likely an important factor in preparing players for matches from both a safety and performance perspective. The high density and intensity of contact training, during which players are under fatigue, might lead to declining proficiency in technique [27–29] and an increase in the probability of experiencing HAEs. The highest risk of tackle concussions to ball-carriers is observed during head-to-head contact [30, 31], whilst it is high for tacklers when their head contacts the ball carrier’s knee, hip, or a teammates head [30]. In rugby union, high tackles were associated with an increased HAE propensity and magnitude [32]. Furthermore, the incidence of HAEs was 182% higher in the final phase of the pre-season, when training is often structured similarly to an in-season session as friendly matches are introduced, compared to the first phase. Comparable HAE incidence at this stage of pre-season compared to in-season training [13] might reflect practitioner’s and coach’s success in replicating in-season training demands in the latter stages of pre-season and successful preparation for competition. Whilst this study did not measure the level of contact intensity during pre-season training sessions and drills, the probable increased focus of controlled- and full-contact training during the middle three and last three weeks of the pre-season coincides with an observed increase in overall training session HAE incidence rates during the same periods.

Coaches have a relatively high degree of control over training activities, and this is a modifiable environment where they can prescribe contact training activities for individual players. Therefore, training can be seen as a target for injury prevention [16]. However, the incidence of HAEs in the pre-season is very low compared to matches [10, 11]. Contact training and conditioning is required during pre-season to physically and mentally prepare players for competition. Consequently, some degree of HAE exposure is likely inevitable and unavoidable. Population-level contact training restrictions would likely have minimal reductions in the absolute count of HAEs at any magnitude threshold [33] and may have a detrimental impact on performance and readiness. A balance between preparing players efficiently for the demands of competition, whilst minimising HAE exposure, likely requires highly individualised player management initiatives [34].

The present study complements other research quantifying elite men’s population-level average exposure to HAEs during matches [10, 11] and training [11, 13] to give a holistic understanding of their accumulation throughout a season. The findings of this study could similarly be used to benchmark against individual player comparisons. This could support practitioners, and governing bodies identify players who accumulate a higher number of HAEs and where (matches, in-season training, pre-season training). This might inform more effective population-level player welfare polices beyond simply restricting contact training duration [33] for example individual player management strategies within club practices. Future research should look at HAE accumulation during matches and training throughout the season, to identify players with greater exposure relative to the population average.

## Limitations

This is the first study to quantify HAEs during pre-season rugby training. However, it is not without its limitations. The results should be interpreted with caution due to the small sample size. There was a total of 244 player-training session exposures from only three (out of ten) teams in the league. From these three teams, more than half of the players who consented to take part did not wear their iMG during a pre-season training session. It is unknown whether players did not participate in training during the pre-season period (e.g., on international duty, extended leave or injured), or whether players opted to not wear their iMG. The proportion of players within sampled clubs adhering to iMG wearing during training HAE studies was similar to that sampled during the in-season [13]. Whilst the prospective observational cohort study design allowed the direct calculation of incidence rates during pre-season training, this study did not explore differences between levels of contact training. The proportion of training assigned to each level of contact training likely varies between teams with different coaching philosophies, including those who did not take part in the study. Consequently, it should not be assumed that all teams spend the same time in each level of contact training as those who took part in this study during these periods of the pre-season. As such, the accumulation of HAEs at different clubs within a pre-season training session might differ.

Finally, the inherent limitations of iMGs should be recognised. The HAE exposure reported during the pre-season is likely an underestimation due to the potential linear acceleration threshold bias [9, 35]. True in-vivo HAEs at the head’s centre of gravity exceeding 8 g are not recorded if the head does not meet this threshold at the anatomical location of the accelerometer embedded within the iMG. The extent of Type II error in the HAE dataset might be high, given the linear acceleration bias’s influence is greater for lower magnitude HAEs < 10 g (~ 75%) [35], which is what appeared to be observed in training. Additionally, it was assumed that players wore their iMG for all their training drill involvements if they recorded at least one period of iMG time on teeth in its duration, based on findings in previous research in which it has also been applied [11, 13]. This assumption might not apply to players who did not wear their iMG during these studies. Finally, this study only examined peak head kinematics calculated over 50 ms and therefore did not consider pulse duration, which might have greater influence over brain strain [36].

## Conclusion

This is the first study to quantify the incidence of HAEs in elite men’s rugby pre-season training. Similarly to what has been reported during in-season rugby training, the incidence and magnitude of HAEs in pre-season training were low compared to matches. Whilst the incidence of HAEs increased as pre-season progressed for forwards, it remained consistent for backs. The higher HAE incidence for forwards during the second three weeks of the season compared to the first is likely due to the increase in contact training density and intensity. Future work should aim to identify players who accumulate more HAEs in matches and training compared to the rest of the population, and why. This study completes a holistic understanding of the population’s average exposure to HAE throughout a rugby union season, which can support HAE projection calculations and be used to benchmark individual exposure against.

## Data Availability

All data relevant to the study are included in the article or uploaded as supplementary information. Anonymised data should be available upon reasonable request.

## References

[1] Roberts SP, Trewartha G, Higgitt RJ, et al. The physical demands of elite English rugby union. J Sports Sci. 2008;26(8):825–33. 18569548 10.1080/02640410801942122

[2] Kuo C, Patton D, Rooks T, et al. On-field deployment and validation for wearable devices. Ann Biomed Eng. 2022;50(11):1372–88. 35960418 10.1007/s10439-022-03001-3 PMC9652208

[3] Hume PA, Theadom A, Lewis GN, et al. A comparison of cognitive function in former rugby union players compared with former non-contact-sport players and the impact of concussion history. Sports Med. 2017;47:1209–20. 27558141 10.1007/s40279-016-0608-8

[4] Evans LJ, O’Brien WT, Spitz G, et al. Associations between instrumented mouthguard-measured head acceleration events and post-match biomarkers of astroglial and axonal injury in male amateur Australian football players. Sports Med. 2025;55(4):1037–49. 39562417 10.1007/s40279-024-02138-6 PMC12011967

[5] Graham NS, Zimmerman KA, Hain J, et al. Biomarker evidence of neurodegeneration in mid-life former rugby players. Brain. 2025. 10.1093/brain/awaf152. 40602789 10.1093/brain/awaf152 PMC12316018

[6] Parker TD, Hain JA, Rooney EJ, et al. Brain health concerns in former rugby players: clinical and cognitive phenotypes. Brain. 2025. 10.1093/brain/awae416. 40602788 10.1093/brain/awae416 PMC12316010

[7] Russell ER, Mackay DF, Lyall D, et al. Neurodegenerative disease risk among former international rugby union players. J Neurol Neurosurg Psychiatry. 2022;93(12):1262–8. 36195436 10.1136/jnnp-2022-329675 PMC9669247

[8] Anns F, Quarrie KL, Milne BJ, et al. Neurodegenerative diseases in male former first-class New Zealand rugby players. Sports Med. 2025;4:1–6. 10.1007/s40279-025-02299-y PMC12982275 40906013

[9] Tooby J, Till K, Gardner A, et al. When to pull the trigger: conceptual considerations for approximating head acceleration events using instrumented mouthguards. Sports Med. 2024;54(6):1361–9. 38460080 10.1007/s40279-024-02012-5 PMC11239719

[10] Tooby J, Woodward J, Tucker R, et al. Instrumented mouthguards in elite-level men’s and women’s rugby union: the incidence and propensity of head acceleration events in matches. Sports Med. 2024;54(5):1327–38. 37906425 10.1007/s40279-023-01953-7 PMC11127838

[11] Roe G, Sawczuk T, Tooby J, et al. Training and match-related head acceleration events in top level domestic senior women’s and men’s rugby union: a multi-league instrumented mouthguard study. Scand J Med Sci Sports. 2024;34(10):e14744. 39428738 10.1111/sms.14744

[12] Parmley J, Weaving D, Whitehead S, et al. The incidence of head acceleration events during pitch-based training and match play in professional men’s rugby league. Scand J Med Sci Sports. 2025;35(11):e70156. 41204748 10.1111/sms.70156 PMC12595519

[13] Hudson S, Tooby J, Roe G, et al. Head acceleration event exposure during elite men’s and women’s rugby union training. Sports Med. 2025. 10.1007/s40279-025-02287-2. 40753282 10.1007/s40279-025-02287-2 PMC12913257

[14] Hudson S, Williams S, Starling L, et al. Training concussions in men’s English Premiership rubgy union: an eight-season prospective cohort study of 153,243 player-weeks. BJSM. Under review. 10.1136/bjsports-2026-111653 PMC13638317 42562618

[15] West SW, Williams S, Kemp SP, et al. Patterns of training volume and injury risk in elite rugby union: an analysis of 1.5 million hours of training exposure over eleven seasons. J Sports Sci. 2020;38(3):238–47. 31755824 10.1080/02640414.2019.1692415

[16] Williams S, Trewartha G, Kemp SP, et al. Time loss injuries compromise team success in elite rugby union: a 7-year prospective study. Br J Sports Med. 2016;50(11):651–6. 26552415 10.1136/bjsports-2015-094798

[17] Kieffer EE, Begonia MT, Tyson AM, Rowson S. A two-phased approach to quantifying head impact sensor accuracy: in-laboratory and on-field assessments. Ann Biomed Eng. 2020;48:2613–25. 33051745 10.1007/s10439-020-02647-1

[18] Liu Y, Domel AG, Yousefsani SA, et al. Validation and comparison of instrumented mouthguards for measuring head kinematics and assessing brain deformation in football impacts. Ann Biomed Eng. 2020;48:2580–98. 32989591 10.1007/s10439-020-02629-3 PMC9555247

[19] Jones B, Tooby J, Weaving D, et al. Ready for impact? A validity and feasibility study of instrumented mouthguards (iMGs). BJSM. 2022;56(20):1171–9. 10.1136/bjsports-2022-105523 35879022

[20] Bussey MD, Pinfold J, Romanchuk J, Salmon D. Anticipatory head control mechanisms in response to impact perturbations: an investigation of club rugby players with and without a history of concussion injury. Phys Ter Sport. 2023;59:7–16. 10.1016/j.ptsp.2022.11.002 36442352

[21] International Olympic Committee Injury and Illness Epidemiology Consensus Group, Bahr R, Clarsen B, Derman W, et al. International Olympic Committee consensus statement: methods for recording and reporting of epidemiological data on injury and illness in sports 2020 (including the STROBE extension for sports injury and illness surveillance (STROBE-SIIS)). Orthop J Sports Med. 2020;8(2):2325967120902908. 32118084 10.1177/2325967120902908 PMC7029549

[22] Bates D, Mächler M, Bolker B, Walker S, Christen RHB, Singmann H, et al. Fitting linear mixed-effects models using lme4. J Stat Softw. 2015;7(67):1–48.

[23] Brooks M, Bolker B, Kristensen K, Maechler M, Magnusson A, Skaug H, Nielsen A, Berg C, Van Bentham K. glmmTMB: generalized linear mixed models using template model builder. 2017.

[24] Sykes D. Periodization and planning of training for rugby. In: The science of rugby. Routledge; 2014. p. 36–54.

[25] Austin D, Gabbett T, Jenkins D. Repeated high-intensity exercise in professional rugby union. J Sports Sci. 2011;29(10):1105–12. 21756130 10.1080/02640414.2011.582508

[26] Di Mascio M, Bradley PS. Evaluation of the most intense high-intensity running period in English FA premier league soccer matches. J Strength Cond Res. 2013;27(4):909–15. 22652921 10.1519/JSC.0b013e31825ff099

[27] Gamble P. A skill-based conditioning games approach to metabolic conditioning for elite rugby football players. J Strength Cond Res. 2004;18(3):491–7. 15320667 10.1519/1533-4287(2004)18<491:ASCGAT>2.0.CO;2

[28] Tierney GJ, Denvir K, Farrell G, Simms CK. The effect of tackler technique on head injury assessment risk in elite rugby union. Med Sci Sports Exerc. 2018;50(3):603–8. 29049096 10.1249/MSS.0000000000001461

[29] Davidow D, Redman M, Lambert M, et al. The effect of physical fatigue on tackling technique in rugby union. J Sci Med Sport. 2020;23(11):1105–10. 32359940 10.1016/j.jsams.2020.04.005

[30] Stokes K, Locke D, Roberts S, et al. Tackle characteristics associated with concussion in elite men’s rugby union: unpicking the differences between tacklers and ball-carriers. BMJ Open Sport Exerc Med. 2025. 10.1136/bmjsem-2025-002612. 40766043 10.1136/bmjsem-2025-002612 PMC12323531

[31] Cross MJ, Tucker R, Raftery M, et al. Tackling concussion in professional rugby union: a case–control study of tackle-based risk factors and recommendations for primary prevention. Br J Sports Med. 2019;53(16):1021–5. 29021244 10.1136/bjsports-2017-097912

[32] Owen C, Roe G, Tooby J, et al. Evaluating the probability of head acceleration events in elite men’s and women’s rugby union match-play: the impact of tackle height and body position. Sports Med. 2025;55(10):2641. 40335879 10.1007/s40279-025-02241-2 PMC12513871

[33] Sawczuk T, Roe G, Tooby J, et al. The effect of changing weekly contact training duration beyond current guidelines on head acceleration events in rugby union. Sports Med. 2025;27:1–13. 10.1007/s40279-025-02359-3 PMC13124761 41298988

[34] Stokes K, Jones B, Bennett M, et al. Returning to play after prolonged training restrictions in professional collision sports. Int J Sports Med. 2020;41(13):895–911. 32483768 10.1055/a-1180-3692 PMC7799169

[35] Wang T, Kenny R, Wu LC. Head impact sensor triggering bias introduced by linear acceleration thresholding. Ann Biomed Eng. 2021;49(12):3189–99. 34622314 10.1007/s10439-021-02868-y

[36] Gabler LF, Crandall JR, Panzer MB. Development of a second-order system for rapid estimation of maximum brain strain. Ann Biomed Eng. 2019;47(9):1971–81. 30515603 10.1007/s10439-018-02179-9

